# Supplementary material for: A scoping review of Youth Mental Health First Aid for adolescents in school, community, and healthcare settings
Source: PLOS Ment Health. 2026 Jan 29;3(1):e0000549. doi: 10.1371/journal.pmen.0000549 (PMC12854427; doi:10.1371/journal.pmen.0000549)
Supplement: S1 Appendix — (PDF) [file pmen.0000549.s001.pdf]

Appendix I: Search Strategy

MEDLINE (via PubMed)

**Language:** English	
**#**	**Query**
1	(“Youth Mental Health First Aid”) OR (“YMHFA”) OR (“ALGEE”) OR (“National Council for Mental Wellbeing”)
2	(schools OR “youth centers” OR “after-school programs” OR “community program*” OR “healthcare setting*” OR clinic* OR hospital* OR “Online training*” OR e-learning OR “Digital mental health” OR “rural communit*” OR “underserved population” OR “culturally diverse communit*”)
3	(adolescen* OR youth OR teen* OR student* OR educator* OR teacher* OR counselor* OR “school staff” OR “healthcare provider*” OR “mental health professional*” OR nurse* OR therapist* OR psychologist* OR coach* OR “youth worker*” OR caregiver* OR parent* OR “peer support” OR “peer mentor” OR “student mentor”)
4	((“Youth Mental Health First Aid”) OR (“YMHFA”) OR (“ALGEE”) OR (“National Council for Mental Wellbeing”)) AND ((((((((((((schools) OR (“youth centers”)) OR (“after-school programs”)) OR (“community program*”)) OR (“healthcare setting*”)) OR (clinic*)) OR (hospital*)) OR (“Online training*”)) OR (e-learning)) OR (“Digital mental health”)) OR (“rural communit*”)) OR (“underserved population”)) OR (“culturally diverse communit*”))) AND ((((((((((((((((((((adolescen*) OR (youth)) OR (teen*)) OR (student*)) OR (educator*)) OR (teacher*)) OR (counselor*)) OR (“school staff”)) OR (“healthcare provider*”)) OR (“mental health professional*”)) OR (nurse*)) OR (therapist*)) OR (psychologist*)) OR (coach*)) OR (“youth worker*”)) OR (caregiver*)) OR (parent*)) OR (“peer support”)) OR (“peer mentor”)) OR (“student mentor”))

EMBASE

**Language:** English	
**#**	**Query**
1	('youth mental health first aid' OR ymhfa OR algee OR 'national council for mental wellbeing') AND ('schools'/exp OR 'schools' OR 'youth centers' OR 'after-school programs' OR 'community program*' OR 'healthcare setting*' OR clinic* OR hospital* OR 'online training*' OR 'e learning'/exp OR 'e learning' OR 'digital mental health'/exp OR 'digital mental health' OR 'rural communit*' OR 'underserved population' OR 'culturally diverse communit*') AND ('adolescen*' OR 'youth'/exp OR youth OR teen* OR student* OR educator* OR teacher* OR counselor* OR 'school staff'/exp OR 'school staff' OR 'healthcare provider*' OR 'mental health professional*' OR nurse* OR therapist* OR psychologist* OR coach* OR 'youth worker*' OR caregiver* OR parent* OR 'peer support'/exp OR 'peer support' OR 'peer mentor' OR 'student mentor')

PsychINFO (via EBSCOhost)

**Language:** English	
**#**	**Query**
1	("Youth Mental Health First Aid" OR YMHFA OR ALGEE OR "National Council for Mental Wellbeing") AND ("schools" OR "youth centers" OR "after-school programs" OR "community program*" OR "healthcare setting*" OR clinic* OR hospital* OR "Online training*" OR e-learning OR "Digital mental health" OR "rural communit*" OR "underserved population" OR "culturally diverse communit*") AND (adolescen* OR youth OR teen* OR student* OR educator* OR teacher* OR counselor* OR "school staff" OR "healthcare provider*" OR "mental health professional*" OR nurse* OR therapist* OR psychologist* OR coach* OR "youth worker*" OR caregiver* OR parent* OR "peer support" OR "peer mentor" OR "student mentor")

ERIC (via Institute of Education Sciences)

**Language:** English	
**#**	**Query**
1	("Youth Mental Health First Aid" OR YMHFA OR ALGEE OR "National Council for Mental Wellbeing") AND (schools OR "youth centers" OR "after-school programs" OR "community program*") AND (adolescen* OR youth OR teen* OR student* OR educator* OR teacher* OR counselor* OR "school staff")

CINAHL (via EBSCOhost)

**Language:** English	
**#**	**Query**
1	("Youth Mental Health First Aid" OR YMHFA OR "ALGEE" OR "National Council for Mental Wellbeing") AND ("schools" OR "youth centers" OR "after-school programs" OR "community program*" OR "healthcare setting*" OR clinic* OR hospital* OR "Online training*" OR e-learning OR "Digital mental health" OR "rural communit*" OR "underserved population" OR "culturally diverse communit*") AND (adolescen* OR youth OR teen* OR student* OR educator* OR teacher* OR counselor* OR "school staff" OR "healthcare provider*" OR "mental health professional*" OR nurse* OR therapist* OR psychologist* OR coach* OR "youth worker*" OR caregiver* OR parent* OR "peer support" OR "peer mentor" OR "student mentor")

SCOPUS

**Language:** English	
**#**	**Query**
1	(TITLE-ABS-KEY(((((("Youth Mental Health First Aid") OR (ymhfa)) OR (algee)) OR ("National Council for Mental Wellbeing")) AND ((((((((((((schools) OR ("youth centers")) OR ("after-school programs")) OR ("community program*")) OR ("healthcare setting*")) OR (clinic*)) OR (hospital*)) OR ("Online training*")) OR (e-learning)) OR ("Digital mental health")) OR ("rural communit*")) OR ("underserved population")) OR ("culturally diverse communit*"))) AND (((((((((((((((((((adolescen*) OR (youth)) OR (teen*)) OR (student*)) OR (educator*)) OR (teacher*)) OR (counselor*)) OR ("school staff")) OR ("healthcare provider*")) OR ("mental health professional*")) OR (nurse*)) OR (therapist*)) OR (psychologist*)) OR (coach*)) OR ("youth worker*")) OR (caregiver*)) OR (parent*)) OR ("peer support")) OR ("peer mentor")) OR ("student mentor"))) AND (LIMIT-TO (LANGUAGE,"English")))
