# Supplementary material for: A scoping review of Youth Mental Health First Aid for adolescents in school, community, and healthcare settings
Source: PLOS Ment Health. 2026 Jan 29;3(1):e0000549. doi: 10.1371/journal.pmen.0000549 (PMC12854427; doi:10.1371/journal.pmen.0000549)
Supplement: S2 Appendix — (PDF) [file pmen.0000549.s002.pdf]

Extraction questions

After completing each article extraction, you'll be asked if you wish to continue extracting another source. Selecting 'Yes' will automatically restart this survey for your next source. Selecting 'No' will complete your data extraction session.

Any questions or issues with survey should be directed to Irfan - ialam@olemiss.edu

Article number

Enter author names exactly as listed in the article or source, following the format: "Last name Initial(s)" (e.g., Smith J, Doe AB).

Enter the complete title of the article or document exactly as it appears in the publication.

Provide the publication year (e.g., 2022)

Enter the country or geographic location where the study was conducted. If multiple countries, list all clearly.

- ○ United States
- ○ International (please specify country)

Choose the study design or type of source (select all that

apply)

- [ ] Quantitative research study
- [ ] Qualitative research study
- [ ] Mixed method research study
- [ ] Systematic review / scoping review
- [ ] Grey literature (reports, policies, theses, dissertations)
- [ ] Unsure
- [ ] Other (please specify)

Secondary classification (optional)

- [ ] Randomized controlled trial (RCT)
- [ ] Quasi-experimental study
- [ ] Observational study (e.g., cohort, cross-sectional)
- [ ] Case study
- [ ] Program evaluation
- [ ] Other (please specify)

Indicate all participant groups involved in or targeted by the study (select all applicable)

- [ ] Adolescents (12–18 years)
- [ ] Educators (teachers, school staff)
- [ ] School counselors
- [ ] Healthcare providers (e.g., nurses, doctors, psychologists)
- [ ] Coaches or sports staff
- [ ] Caregivers or parents
- [ ] Youth workers or community workers
- [ ] Other
- [ ] Not applicable

Select the context(s) in which YMHFA was implemented or studied (select all applicable)

- [ ] Schools (middle or high schools)
- [ ] Community (youth centers, clubs, extracurricular settings)
- [ ] Clinical/Healthcare settings (clinics, hospitals, counseling services)
- [ ] Online or digital settings
- [ ] Other
- [ ] Not applicable

Select all aspects of YMHFA specifically addressed in the study (select all applicable)

- ☐ Implementation methods or strategies
- ☐ Effectiveness evaluations (e.g., outcomes of the intervention)
- ☐ Changes in knowledge, attitudes, or behaviors related to mental health
- ☐ Confidence or self-efficacy of participants
- ☐ Stigma reduction
- ☐ Mental health literacy
- ☐ Other ____________________
- ☐ Not applicable

Indicate which outcomes related to YMHFA were reported in this paper. Check all that are reported:

- ☐ Increased mental health literacy
- ☐ Improved confidence or preparedness
- ☐ Increased willingness to intervene
- ☐ Reported behavior changes (engagement in mental health discussions and actions)
- ☐ Reduced mental health stigma
- ☐ Reported positive attitude changes

- [ ] Other (please specify) ____________________
- [ ] Not applicable

Indicate which facilitators of YMHFA implementation were noted in this paper. Check all that are reported:

- [ ] Supportive leadership or administration
- [ ] Availability of funding or resources
- [ ] High-quality or evidence-based training (e.g., ALGEE model)
- [ ] Cultural relevance or adaptations
- [ ] Strong school-family or community partnerships
- [ ] Integration into existing school or organizational systems
- [ ] Instructor involvement or certification support
- [ ] Use of flexible or blended training formats
- [ ] Other (please specify) ____________________
- [ ] Not applicable

Indicate which barriers to YMHFA implementation were reported in this paper. Check all that are reported:

- [ ] Lack of funding or resources

- [ ] Time constraints or scheduling difficulties
- [ ] Staff resistance or low motivation
- [ ] Cultural or language barriers
- [ ] Persistent mental health stigma
- [ ] Limited availability of mental health professionals
- [ ] Technological or internet access issues (for virtual training)
- [ ] Inadequate post-training support or follow-up
- [ ] Other (please specify) ____________
- [ ] Not applicable

Would you recommend this article for the scoping review?

- ( ) Yes
- ( ) No
- ( ) Not sure

Additional Comments or Notes (Optional)

____

Additional extraction

Would you like to extract data from another article/source?

- ○ Yes
- ○ No

Powered by Qualtrics
