# Supplementary material for: A scoping review of Youth Mental Health First Aid for adolescents in school, community, and healthcare settings
Source: PLOS Ment Health. 2026 Jan 29;3(1):e0000549. doi: 10.1371/journal.pmen.0000549 (PMC12854427; doi:10.1371/journal.pmen.0000549)
Supplement: S3 Appendix — (PDF) [file pmen.0000549.s003.pdf]

University of Mississippi

eGrove

Faculty and Student Publications

3-31-2025

A Scoping Review Protocol of Youth Mental Health First Aid for Adolescents in School, Community, and Healthcare Settings

Irfanul Alam
University of Mississippi

Marie Barnard
University of Mississippi

Jessica Osbourne
University of Mississippi

Divya Chandran Geetha Kumari
University of Mississippi

Clyde King Jr.
University of Mississippi

See next page for additional authors

Follow this and additional works at: https://egrove.olemiss.edu/public-health-facpubs

Recommended Citation

Alam, Irfanul; Barnard, Marie; Osbourne, Jessica; Kumari, Divya Chandran Geetha; King, Clyde Jr.; Ford, M. Allison; and Allen, Hannah K., "A Scoping Review Protocol of Youth Mental Health First Aid for Adolescents in School, Community, and Healthcare Settings" (2025). *Faculty and Student Publications*. 1.
https://egrove.olemiss.edu/public-health-facpubs/1

This Report is brought to you for free and open access by eGrove. It has been accepted for inclusion in Faculty and Student Publications by an authorized administrator of eGrove. For more information, please contact egrove@olemiss.edu.

Authors

Irfanul Alam, Marie Barnard, Jessica Osbourne, Divya Chandran Geetha Kumari, Clyde King Jr., M. Allison Ford, and Hannah K. Allen

This report is available at eGrove: https://egrove.olemiss.edu/public-health-facpubs/1

A Scoping Review Protocol of Youth Mental Health First Aid for Adolescents in School, Community, and Healthcare Settings.

Authors

Irfanul Alam[1], Marie Barnard[2,3], Jessica Osborne[1], Divya Chandran Geetha Kumari[2], Clyde King, Jr.[2], M. Allison Ford[2], Hannah K. Allen[2,4]

1. Center for Research Evaluation, The University of Mississippi, P.O. Box 1848, University, MS 38677
2. Department of Public Health, The University of Mississippi, P.O. Box 1848, University, MS 38677
3. Department of Pharmacy Administration, The University of Mississippi, P.O. Box 1848, University, MS 38677
4. William Magee Institute for Student Wellbeing, The University of Mississippi, P.O. Box 1848, University, MS 38677

Keywords: adolescent mental health; early intervention; mental health literacy; school-based programs; youth crisis support

Introduction

Youth Mental Health First Aid (YMHFA) is an evidence-based training program designed to enhance mental health literacy, early intervention capabilities, and crisis response skills among individuals who interact with young people. The program is structured around the ALGEE model, which includes assessing for risk of harm, listening without judgment, giving reassurance, encouraging professional help, and promoting self-help strategies. Research suggests that YMHFA significantly improves participants' knowledge, confidence, and willingness to intervene in youth mental health crises (Aakre et al., 2016; Noltemeyer et al., 2019). However, concerns persist regarding long-term behavioral retention and its direct impact on youth mental health outcomes (Sánchez et al., 2021).

YMHFA has been implemented across diverse settings in the United States, including schools, rural communities, and youth-serving organizations. Despite its growing adoption, studies indicate that implementation challenges, such as mental health stigma, limited mental health professionals in rural areas, and logistical barriers, impact program effectiveness (Graves et al., 2024; Haggerty et al., 2019). Conversely, strong institutional support, school-based integration, and community partnerships have been identified as key facilitators that enhance YMHFA sustainability and impact (Boulden & Schimmel, 2024; Reynolds, 2023). While existing research provides valuable insights, there is a need for a comprehensive review to synthesize and map findings across studies, particularly in identifying gaps and best practices.

A preliminary search of MEDLINE, the Cochrane Database of Systematic Reviews, and JBI Evidence Synthesis was conducted, and no current or ongoing systematic reviews or scoping reviews on Youth Mental Health First Aid (YMHFA) were identified. While studies have evaluated the effectiveness and implementation of YMHFA, no comprehensive synthesis exists to map the extent of the literature on this topic. This scoping review aims to fill that gap by identifying, categorizing, and examining existing evidence regarding YMHFA, its implementation, effectiveness, barriers, and facilitators.

Key Terms and Operational Definitions

For this review, several key terms require clarification:

1. Youth Mental Health First Aid (YMHFA): A structured training program designed to equip adults with the knowledge and skills to support youth experiencing mental health challenges or crises (Geierstanger et al., 2024).
2. Mental health literacy: The knowledge and beliefs about mental health disorders that aid in their recognition, management, and prevention.
3. Implementation: The process by which YMHFA training is adopted, delivered, and sustained within various community and institutional settings.
4. Effectiveness: The extent to which YMHFA achieves its intended outcomes, including increased knowledge, improved crisis response, and behavioral changes among participants.
5. Barriers and Facilitators: Factors that hinder or enhance the successful implementation and effectiveness of YMHFA programs.

This scoping review will operationalize these terms based on existing literature and will categorize study findings accordingly.

Justification for a Scoping Review

A scoping review methodology was selected as the most suitable approach for this study due to the broad and exploratory nature of the research question. In contrast to systematic reviews, which aim to answer specific questions about intervention effectiveness, scoping reviews are particularly useful for mapping the extent, range, and nature of evidence available on a given topic (Arksey & O'Malley, 2005; Levac et al., 2010). Considering the diversity of study designs in YMHFA research—including quantitative, qualitative, and mixed-methods approaches—a scoping review enables a comprehensive synthesis of findings without applying restrictive inclusion criteria.

Furthermore, YMHFA research encompasses multiple disciplines, such as education, psychology, public health, and community engagement. A scoping review can synthesize findings from these various perspectives, offering a comprehensive understanding of program implementation and effectiveness. In addition, this review will help pinpoint existing research gaps, guiding future studies and policy recommendations.

Preliminary Evidence

Preliminary evidence points to a substantial body of literature on YMHFA, including studies assessing its effectiveness among educators, parents, and youth-serving professionals (Chehaib et al., 2023; Marsico et al., 2022; Noltemeyer et al., 2019). Research also reveals significant implementation challenges, especially in rural and culturally diverse communities, where stigma and logistical barriers obstruct program adoption (Graves et al., 2024; Henderson et al., 2024). These findings underscore the need for a structured synthesis to consolidate existing knowledge and inform future program improvements.

Review Objectives

The goal of this scoping review is to evaluate the scope of the literature on YMHFA, concentrating on its implementation, effectiveness, barriers, and facilitators. Specifically, this review intends to:

1. Identify studies and describe reports of YMHFA implementation and effectiveness.
2. Explore key facilitators and barriers affecting YMHFA adoption in different settings.
3. Highlight research gaps and areas for future investigation.

This review will map the existing evidence, contributing to a more comprehensive understanding of YMHFA's role in youth mental health support, inform strategies for optimizing its impact, and provide information that can support recommendations for future program implementations.

Review questions

What literature currently exists on Youth Mental Health First Aid (YMHFA) for adolescents aged 12 to 18? Within that literature, what are the primary findings related to implementation and effectiveness broadly?

Inclusion criteria

Participants

This scoping review will include studies involving adolescents aged 12–18 and those involving adults who directly support or interact with adolescents within this age range, including but not limited to educators, school counselors, healthcare providers, coaches, caregivers, and youth workers. This review will also include studies where adolescents serve as peer supporters within YMHFA programs, provided they align with the primary focus on adolescent mental health intervention. Studies will not be excluded based on gender, ethnicity, race, socioeconomic status, or specific health conditions. However, studies exclusively focused on adults or children younger than 12 without explicit relevance to the adolescent age group (12–18) will be excluded.

Concept

The concept under investigation is Youth Mental Health First Aid (YMHFA), a structured educational intervention aimed at teaching individuals how to recognize signs and symptoms of adolescent mental health issues, provide initial support, and direct adolescents toward appropriate professional help or self-care strategies. Studies eligible for inclusion must clearly address one or more elements of YMHFA, including its implementation processes or strategies, evaluations of effectiveness, feasibility, or impact, or outcomes measured through changes in knowledge, attitudes, confidence, skills, behaviors, or other impacts specifically related to adolescent mental health. Studies solely examining general Mental Health First Aid (MHFA) without a clear adaptation or application for adolescents aged 12–18 will be excluded.

Context

This review will encompass studies conducted in real-world or practical contexts relevant to adolescents, including educational settings (e.g., schools), community environments (e.g., youth centers, after-school programs), healthcare locations (e.g., clinics, hospitals), and virtual or online platforms. The geographic context is not restricted, and studies conducted internationally will be considered. Literature examining cultural, racial or ethnic, gender-specific, socioeconomic, or sub-cultural factors pertinent to the implementation, delivery, or outcomes of YMHFA programs for adolescents will be explicitly included. Studies that are purely theoretical, opinion-based, or

commentary lacking empirical data, or those that exclusively focus on adult contexts without direct relevance to adolescents will be excluded.

Types of sources

This review will examine various sources of evidence to thoroughly map the existing literature related to Youth Mental Health First Aid (YMHFA) for adolescents aged 12 to 18 years. Specifically, studies with experimental and quasi-experimental designs—including randomized controlled trials (RCTs), non-randomized controlled trials, before-and-after studies, and interrupted time-series studies—will be included. Analytical observational studies, such as prospective and retrospective cohort studies, case-control studies, and analytical cross-sectional studies, will also qualify for inclusion. Furthermore, descriptive observational designs, including descriptive cross-sectional studies, case series, and individual case reports, will be reviewed to provide broader descriptive insights. Qualitative studies, including those using thematic analysis and other inductive methodologies, will be included to capture lived experiences, perceptions, and contextual factors related to YMHFA implementation and outcomes.

Systematic reviews and other knowledge syntheses (e.g., scoping reviews, rapid reviews, and evidence maps) that align with the inclusion criteria will also be considered. Additionally, gray literature sources will include policy documents, government or NGO reports, dissertations, theses, conference proceedings, and unpublished evaluations from organizations involved in YMHFA implementation (e.g., WHO, Mental Health First Aid International, SAMHSA, NIMH).

Methods

The proposed scoping review will be conducted according to the Joanna Briggs Institute methodology for scoping reviews (JBI; Aromataris et al., 2024) and Preferred Reporting Items for Systematic Reviews and Meta Analyses extension for Scoping Reviews (PRISMA-ScR; Tricco et al., 2018). The protocol will be deposited in the University of Mississippi's open-access online repository, eGrove.

Search strategy

The search strategy will aim to locate both published and unpublished studies. A three-step search strategy will be utilized in this review. First, an initial limited search of MEDLINE (PubMed) was undertaken to identify articles on the topic. The text words contained in the titles and abstracts of relevant articles and the index terms used to describe the articles were used to develop a full search strategy for reporting the names of the relevant databases/information sources *(see Appendix I*). The search strategy, including all identified keywords and index terms, will be adapted for each included database and/or information source. The reference list of all included sources of evidence will be screened for additional studies. Only studies published in the English language and available in full text will be included in the review. No date limitations will be applied.

Study/Source of evidence selection

After the search, all identified citations will be compiled and uploaded into Zotero, with duplicates removed. Following a pilot test, titles and abstracts will be screened by two or more independent reviewers to assess them against the inclusion criteria for the review. The full texts of all identified articles will be retrieved for the next stage of the review. Two or more independent reviewers will evaluate selected citations' full texts in detail against the inclusion criteria. Reasons for excluding sources of evidence at the full-text stage that do not meet the inclusion criteria will be documented and reported in the scoping review. Any disagreements that arise between the reviewers at each

stage of the selection process will be resolved through discussion or with the help of an additional reviewer. The search results and the study inclusion process will be comprehensively reported in the final scoping review and presented in a PRISMA flow diagram (Page et al., 2021).

Data extraction

A data extraction form will be created in Qualtrics. The form will be piloted and updated as needed. Data will be extracted from the papers identified for full text review by two or more independent reviewers. The extracted data will include specific details about the participants, concepts, context, study methods, reported facilitators and barriers to implementation, as well as program effectiveness and outcome data.

A draft extraction form is provided (*see Appendix II*). The data extraction tool will be modified and revised as necessary during the process of extracting data from each included evidence source. Modifications will be detailed in the scoping review. Any disagreements that arise between the reviewers will be resolved through discussion or with an additional reviewer. If appropriate, authors of papers will be contacted to request missing or additional data.

Data analysis and presentation

Data will be organized and tabulated by concept, context, study methods, reported facilitators and barriers to implementation, and program effectiveness and outcome data for each included article. A narrative summary will accompany the tabulated and charted results, describing how they relate to the review question and objectives.

Acknowledgments

The authors would like to acknowledge the support provided by the Southern Mental Health Alliance for administrative and logistical assistance in conducting this research.

Funding

This research was funded by the Office of National Drug Control Policy (ONDCP), Award Numbers #CDS9923G0006 and #CDS9924G0017 (PI: H.K. Allen). The funding agency had no role in the study design, data collection, data analysis, interpretation of findings, or decision to submit this manuscript for publication.

Author contributions

I.A. coordinated the overall execution of the scoping review, conducted the literature search, and synthesized key findings. Additionally, I.A. led the drafting of the manuscript, integrated feedback from co-authors, managed revisions, and prepared the final submission of the manuscript. M.B provided study design and methodological guidance, participated in data extraction, analysis, and interpretation, and manuscript review and editing. J.O. supported data analysis and interpretation and assisted with manuscript review and editing. D.C.G participated in abstract reviews, full text screenings of articles, and the review and editing of the manuscript. C.K.J. contributed to the abstract review and full-text screenings of articles. M.A.F is the lead investigator for this project and contributed to the review and editing of the manuscript. H.A. oversaw acquisition of funding support

for the project leading to this publication and contributed to the review and editing of the manuscript.

Conflicts of interest

There is no conflict of interest in this project.

References

Aakre, J. M., Lucksted, A., & Browning-McNee, L. A. (2016). Evaluation of Youth Mental Health First Aid USA: A program to assist young people in psychological distress. *Psychological Services, 13*(2), 121.

Arksey, H., & O'malley, L. (2005). Scoping studies: towards a methodological framework. *International journal of social research methodology*, *8*(1), 19-32.

Aromataris, E., Lockwood, C., Porritt, K., Pilla, B., & Jordan, Z. (Eds.). (2024). *Scoping reviews*. In *JBI Manual for Evidence Synthesis*. JBI. Retrieved from https://synthesismanual.jbi.global

Boulden, R., & Schimmel, C. (2024). Addressing the rural youth mental health crisis through Youth Mental Health First Aid. *The Rural Educator, 45*(2), 61–67. https://doi.org/10.55533/2643-9662.1413

Chehaib, H., Rodríguez-Campos, L., & Todd, A. (2023). Evaluation of a school-based program designed to improve mental health in children: A collaborative approach. *School Community Journal, 33*(1), 229–250.

Geierstanger, S., Yu, J., Saphir, M., & Soleimanpour, S. (2024). Youth Mental Health First Aid Training: Impact on the ability to recognize and support youth needs. *The Journal of Behavioral Health Services & Research, 51*(4), 588–598. https://doi.org/10.1007/s11414-024-09893-4

Graves, J. M., Abshire, D. A., Koontz, E., & Mackelprang, J. L. (2024). Identifying challenges and solutions for improving access to mental health services for rural youth: Insights from adult community members. *International Journal of Environmental Research and Public Health, 21*, 725. https://doi.org/10.3390/ijerph21060725

Haggerty, D., Carlson, J. S., McNall, M., Lee, K., & Williams, S. (2019). Exploring Youth Mental Health First Aider training outcomes by workforce affiliation: A survey of Project AWARE participants. *School Mental Health, 11*(4), 345–356. https://doi.org/10.1007/s12310-018-9300-5

Henderson, M. M., Bowie-Viverette, A. C., Coronado, R., Gomez, R. J., Cuevas, M., Healy, D., O'Donnell, K., & Martinez-Gollar, L. (2024). Esperanza: Increasing equity and access in mental health services for adolescents. *School Social Work Journal, 48*(2), Spring 2024.

Levac, D., Colquhoun, H., & O'brien, K. K. (2010). Scoping studies: advancing the methodology. *Implementation science*, *5*, 1-9.

Marsico, K. F., Wang, C., & Liu, J. (2022). Effectiveness of Youth Mental Health First Aid training for parents at school. *Psychology in the Schools, 59*(8), 1701–1716.

Noltemeyer, A., Huang, H., Meehan, C., Jordan, E., Morio, K., Shaw, K., & Oberlin, K. (2019). Youth Mental Health First Aid: Initial outcomes of a statewide rollout in Ohio. *Journal of Applied School Psychology.* https://doi.org/10.1080/15377903.2019.1619645

Page, M. J., McKenzie, J. E., Bossuyt, P. M., Boutron, I., Hoffmann, T. C., Mulrow, C. D., ... & Moher, D. (2021). The PRISMA 2020 statement: an updated guideline for reporting systematic reviews. *bmj*, *372*.

Reynolds, S. (2023). Improving youth access to mental health: An intervention for rural non-profit organizations. *Doctoral Dissertation, Capella University.*

Sánchez, A. M., Latimer, J. D., Scarimbolo, K., von der Embse, N. P., Suldo, S. M., & Salvatore, C. R. (2021). Youth Mental Health First Aid (Y-MHFA) trainings for educators: A systematic review. *School Mental Health, 13*, 1–12. https://doi.org/10.1007/s12310-020-09393-8

Tricco, A. C., Lillie, E., Zarin, W., O'Brien, K. K., Colquhoun, H., Levac, D., ... & Straus, S. E. (2018). PRISMA extension for scoping reviews (PRISMA-ScR): checklist and explanation. *Annals of internal medicine*, *169*(7), 467-473.

Appendix I: Search Strategy

MEDLINE (via PubMed)

**Language:** English	
**#**	**Query**
1	(“Youth Mental Health First Aid”) OR (“YMHFA”) OR (“ALGEE”) OR (“National Council for Mental Wellbeing”)
2	(schools OR “youth centers” OR “after-school programs” OR “community program*” OR “healthcare setting*” OR clinic* OR hospital* OR “Online training*” OR e-learning OR “Digital mental health” OR “rural communit*” OR “underserved population” OR “culturally diverse communit*”)
3	(adolescen* OR youth OR teen* OR student* OR educator* OR teacher* OR counselor* OR “school staff” OR “healthcare provider*” OR “mental health professional*” OR nurse* OR therapist* OR psychologist* OR coach* OR “youth worker*” OR caregiver* OR parent* OR “peer support” OR “peer mentor” OR “student mentor”)
4	((“Youth Mental Health First Aid”) OR (“YMHFA”) OR (“ALGEE”) OR (“National Council for Mental Wellbeing”)) AND ((((((((((((schools) OR (“youth centers”)) OR (“after-school programs”)) OR (“community program*”)) OR (“healthcare setting*”)) OR (clinic*)) OR (hospital*)) OR (“Online training*”)) OR (e-learning)) OR (“Digital mental health”)) OR (“rural communit*”)) OR (“underserved population”)) OR (“culturally diverse communit*”))) AND (((((((((((((((((((adolescen*) OR (youth)) OR (teen*)) OR (student*)) OR (educator*)) OR (teacher*)) OR (counselor*)) OR (“school staff”)) OR (“healthcare provider*”)) OR (“mental health professional*”)) OR (nurse*)) OR (therapist*)) OR (psychologist*)) OR (coach*)) OR (“youth worker*”)) OR (caregiver*)) OR (parent*)) OR (“peer support”)) OR (“peer mentor”)) OR (“student mentor”))

EMBASE

**Language:** English	
**#**	**Query**
1	('youth mental health first aid' OR ymhfa OR algee OR 'national council for mental wellbeing') AND ('schools'/exp OR 'schools' OR 'youth centers' OR 'after-school programs' OR 'community program*' OR 'healthcare setting*' OR clinic* OR hospital* OR 'online training*' OR 'e learning'/exp OR 'e learning' OR 'digital mental health'/exp OR 'digital mental health' OR 'rural communit*' OR 'underserved population' OR 'culturally diverse communit*') AND ('adolescen*' OR 'youth'/exp OR youth OR teen* OR student* OR educator* OR teacher* OR counselor* OR 'school staff'/exp OR 'school staff' OR 'healthcare provider*' OR 'mental health professional*' OR nurse* OR therapist* OR psychologist* OR coach* OR 'youth worker*' OR caregiver* OR parent* OR 'peer support'/exp OR 'peer support' OR 'peer mentor' OR 'student mentor')

PsychINFO (via EBSCOhost)

**Language:** English	
**#**	**Query**
1	("Youth Mental Health First Aid" OR YMHFA OR ALGEE OR "National Council for Mental Wellbeing") AND ("schools" OR "youth centers" OR "after-school programs" OR "community program*" OR "healthcare setting*" OR clinic* OR hospital* OR "Online training*" OR e-learning OR "Digital mental health" OR "rural communit*" OR "underserved population" OR "culturally diverse communit*") AND (adolescen* OR youth OR teen* OR student* OR educator* OR teacher* OR counselor* OR "school staff" OR "healthcare provider*" OR "mental health professional*" OR nurse* OR therapist* OR psychologist* OR coach* OR "youth worker*" OR caregiver* OR parent* OR "peer support" OR "peer mentor" OR "student mentor")

ERIC (via Institute of Education Sciences)

**Language:** English	
**#**	**Query**
1	("Youth Mental Health First Aid" OR YMHFA OR ALGEE OR "National Council for Mental Wellbeing") AND (schools OR "youth centers" OR "after-school programs" OR "community program*") AND (adolescen* OR youth OR teen* OR student* OR educator* OR teacher* OR counselor* OR "school staff")

CINAHL (via EBSCOhost)

**Language:** English	
**#**	**Query**
1	("Youth Mental Health First Aid" OR YMHFA OR "ALGEE" OR "National Council for Mental Wellbeing") AND ("schools" OR "youth centers" OR "after-school programs" OR "community program*" OR "healthcare setting*" OR clinic* OR hospital* OR "Online training*" OR e-learning OR "Digital mental health" OR "rural communit*" OR "underserved population" OR "culturally diverse communit*") AND (adolescen* OR youth OR teen* OR student* OR educator* OR teacher* OR counselor* OR "school staff" OR "healthcare provider*" OR "mental health professional*" OR nurse* OR therapist* OR psychologist* OR coach* OR "youth worker*" OR caregiver* OR parent* OR "peer support" OR "peer mentor" OR "student mentor")

SCOPUS

**Language:** English	
**#**	**Query**
1	(TITLE-ABS-KEY(((((("Youth Mental Health First Aid") OR (ymhfa)) OR (algee)) OR ("National Council for Mental Wellbeing")) AND ((((((((((((schools) OR ("youth centers")) OR ("after-school programs")) OR ("community program*")) OR ("healthcare setting*")) OR (clinic*)) OR (hospital*)) OR ("Online training*")) OR (e-learning)) OR ("Digital mental health")) OR ("rural communit*")) OR ("underserved population")) OR ("culturally diverse communit*"))) AND ((((((((((((((((((adolescen*) OR (youth)) OR (teen*)) OR (student*)) OR (educator*)) OR (teacher*)) OR (counselor*)) OR ("school staff")) OR ("healthcare provider*")) OR ("mental health professional*")) OR (nurse*)) OR (therapist*)) OR (psychologist*)) OR (coach*)) OR ("youth worker*")) OR (caregiver*)) OR (parent*)) OR ("peer support")) OR ("peer mentor")) OR ("student mentor"))) AND (LIMIT-TO (LANGUAGE,"English")))

Appendix II: YMHFA Scoping Review Extraction Survey

Start of Block: Extraction questions

Q1 After completing each article extraction, you'll be asked if you wish to continue extracting another source. Selecting 'Yes' will automatically restart this survey for your next source. Selecting 'No' will complete your data extraction session.

Any questions or issues with survey should be directed to Irfan - ialam@olemiss.edu

Page Break

Q2 Enter author names exactly as listed in the article or source, following the format: "Last name Initial(s)" (e.g., Smith J, Doe AB).

____

Q3 Enter the complete title of the article or document exactly as it appears in the publication.

____

____

____

____

____

Q4 Provide the publication year (e.g., 2022)

____

Page Break

Q5 Enter the country or geographic location where the study was conducted. If multiple countries, list all clearly.

____

Q6 Choose the study design or type of source (select all that apply)

- ☐ Randomized controlled trial (RCT)
- ☐ Non-randomized controlled trial
- ☐ Quasi-experimental (e.g., pre-post, interrupted time-series)
- ☐ Prospective cohort study
- ☐ Retrospective cohort study
- ☐ Analytical cross-sectional study
- ☐ Descriptive cross-sectional study
- ☐ Qualitative research study
- ☐ Systematic review or scoping review
- ☐ Grey literature (reports, policies, theses, dissertations)
- ☐ Other ____
- ☐ Unsure

Q7 Indicate all participant groups involved in or targeted by the study (select all applicable)

- ☐ Adolescents (12–18 years)
- ☐ Educators (teachers, school staff)
- ☐ School counselors
- ☐ Healthcare providers (e.g., nurses, doctors, psychologists)
- ☐ Coaches or sports staff
- ☐ Caregivers or parents
- ☐ Youth workers or community workers
- ☐ Other ____

Q8 Select the context(s) in which YMHFA was implemented or studied (select all applicable)

- ☐ Schools (middle or high schools)
- ☐ Community (youth centers, clubs, extracurricular settings)
- ☐ Clinical/Healthcare settings (clinics, hospitals, counseling services)
- ☐ Online or digital settings
- ☐ Other ____

Q9 Select all aspects of YMHFA specifically addressed in the study (select all applicable)

- ▢ Implementation methods or strategies
- ▢ Effectiveness evaluations (e.g., outcomes of the intervention)
- ▢ Changes in knowledge, attitudes, or behaviors related to mental health
- ▢ Confidence or self-efficacy of participants
- ▢ Stigma reduction
- ▢ Mental health literacy
- ▢ Other ____

Page Break

Q10 Indicate which outcomes related to YMHFA were reported in this paper. Check all that are reported:

- ☐ Increased mental health literacy
- ☐ Improved confidence or preparedness
- ☐ Increased willingness to intervene
- ☐ Reported behavior changes (engagement in mental health discussions and actions)
- ☐ Reduced mental health stigma
- ☐ Reported positive attitude changes
- ☐ Other (please specify) ____

Q11 Indicate which facilitators of YMHFA implementation were noted in this paper. Check all that are reported:

- ☐ Supportive leadership or administration
- ☐ Availability of funding or resources
- ☐ High-quality or evidence-based training (e.g., ALGEE model)
- ☐ Cultural relevance or adaptations
- ☐ Strong school-family or community partnerships
- ☐ Integration into existing school or organizational systems
- ☐ Instructor involvement or certification support
- ☐ Use of flexible or blended training formats
- ☐ Other (please specify) ____

Q12 Indicate which barriers to YMHFA implementation were reported in this paper. Check all that are reported:

- ☐ Lack of funding or resources
- ☐ Time constraints or scheduling difficulties
- ☐ Staff resistance or low motivation
- ☐ Cultural or language barriers
- ☐ Persistent mental health stigma
- ☐ Limited availability of mental health professionals
- ☐ Technological or internet access issues (for virtual training)
- ☐ Inadequate post-training support or follow-up
- ☐ Other (please specify) ____

Page Break

Q13 Additional Comments or Notes (Optional)

____

____

____

____

____

End of Block: Extraction questions

Start of Block: Additional extraction

Q14 Would you like to extract data from another article/source?

- ○ Yes
- ○ No

End of Block: Additional extraction
