# Supplementary material for: A scoping review of Youth Mental Health First Aid for adolescents in school, community, and healthcare settings
Source: PLOS Ment Health. 2026 Jan 29;3(1):e0000549. doi: 10.1371/journal.pmen.0000549 (PMC12854427; doi:10.1371/journal.pmen.0000549)
Supplement: Attachment — Submitted filename: Response to Reviewers.docx [file pmen.0000549.s006.docx]

Manuscript ID: PMEN-D-25-00530
Title: *A scoping review of Youth Mental Health First Aid for adolescents in school, community, and healthcare settings*

Dear Dr. Li,

Thank you for the opportunity to revise our manuscript and for the thoughtful and constructive feedback provided by you and the reviewers. We appreciate the time and care invested in evaluating our work.

We have carefully considered all comments and have undertaken substantial revisions to strengthen the manuscript’s clarity, methodological transparency, and conceptual contribution. Key revisions include a substantially expanded Introduction that more fully contextualizes YMHFA and justifies the use of a scoping review methodology; a reorganized Methods section aligned more clearly with the PCC framework; strengthened Results sections with clearer narrative context; and a revised Discussion that explicitly addresses heterogeneity across study designs, settings, and delivery modalities, as well as gaps related to adolescent-level outcomes.

Detailed, point-by-point responses to all reviewer comments are provided below this letter, with references to specific line numbers in the revised manuscript.

We believe these revisions have significantly improved the manuscript and addressed all concerns raised. We are grateful for the opportunity to revise and resubmit our work and appreciate your consideration.

Sincerely,
Irfanul Alam, PhD
Corresponding Author
On behalf of all co-authors

| **Reviewer Feedback** | **Authors’ Response** |
| --- | --- |
| Reviewer #1: Thank you for the opportunity to review your manuscript. This is a good and timely paper that will add to the literature on youth mental health. However, the manuscript needs some major revisions. Suggestions and comments are attached below. | Thank you for taking the time to provide this valuable feedback. Responses to each comment are below. |
| General comments: 1. The paper is written in passive voice, which makes it difficult to read. | We revised the manuscript to increase use of active voice, particularly in the Methods and Results sections (e.g., descriptions of the search process, screening procedures, and data extraction). Passive constructions were retained only where appropriate for clarity. |
| 2. There are no line numberings for the results section after table 1. Needs to work on it. | Continuous line numbering has been added throughout the revised manuscript file. |
| Abstract: Line 23 - Provide full text for YMHFA rather than acronyms. Provide full text for PRISMA-ScR if possible. | We revised the Abstract to spell out Youth Mental Health First Aid (YMHFA) and removed PRISMA-ScR.  Lines 19-20 |
| Line 25 - Sentence is incomplete | Fixed |
| Introduction: The introduction needs a little more work. Add about two paragraphs to deeply engage with works on YMHFA. What you have now is too shallow. | We substantially revised and expanded the Introduction to provide a deeper and more comprehensive engagement with the YMHFA literature.  In addition, we expanded the discussion of how the YMHFA evidence base has evolved since the 2019 systematic review, explicitly justifying the use of a scoping review methodology to capture emerging implementation-focused, culturally adapted, and virtual or hybrid delivery studies.  Lines 39–74 |
| Line 36 - You mention the program is structured around The Action Plan. What is the action plan? We need context. | Fixed the typo |
| Line 43 - Statement needs a citation. | Added |
| Methods: Line 81 - Use eligibility rather than participants. | Fixed |
| Line 97 to 103 - Has this not been discussed already? Content in this subsection seems to have been discussed earlier. Check the subsection on participants. Perhaps combine them? | We addressed this concern by restructuring the Methods section to more clearly align with the PCC framework. Content related to *who* was eligible for inclusion (adolescents and adults who support adolescents) is now contained within the “Eligibility” subsection, while the “Context” subsection is limited to *where* and *under what conditions* YMHFA was implemented (e.g., settings, platforms, and cultural contexts). This revision was intended to eliminate redundancy and clarify the distinct roles of population and context in the inclusion criteria.  Lines 106–113 and 122–126 |
| Line 104 - Just use "Source" | Fixed |
| Line 105 to 114 - This is same as above. Combine these subsections and rewrite. | We addressed this concern by consolidating content related to eligible study designs and evidence types into a single subsection describing the “Source” of evidence, consistent with the PCC framework. This subsection now captures all eligible quantitative, qualitative, mixed-methods, and evaluation designs, as well as knowledge syntheses and gray literature sources, eliminating redundancy with earlier sections.  Lines 128–139 |
| Line 122 to 128 - Same as above comment. It would be best to have a subsection titled "Inclusion and Exclusion" and put everything about inclusion and exclusion in there. | We addressed this concern by separating inclusion and exclusion *criteria* from the *study selection process*. All eligibility-related content (population, concept, context, and source) is now consolidated within the Eligibility section, consistent with the PCC framework. The “Source Selection” subsection is limited to describing screening and selection procedures (e.g., deduplication, pilot screening, independent review, and discrepancy resolution) and no longer duplicates inclusion or exclusion criteria.  Lines 104–127 and 147-155 |
| Line 131 to 146 - These subsections can be added to the subsection titled "Search Strategy." Maybe a different title after combining would be helpful. | We addressed concerns about redundancy by clarifying the scope of each Methods subsection rather than combining them. The “Search Strategy” subsection is limited to how sources were identified, while “Data Extraction” and “Data Charting and Synthesis” describe post-selection procedures for extracting, organizing, and synthesizing data. This separation follows PRISMA-ScR and JBI guidance and was intended to improve transparency by distinguishing between search, selection, and analytic phases of the review.  Lines 140–176 |
| Results: Needs an introduction. | We added a brief introductory paragraph at the start of the Results section to orient readers to the structure and analytic domains presented.  Lines 210–214 |
| Line 148 to 154 - These two subsections should be in the Methods section and maybe combined with the appropriate subsections. They do not seem to belong in the Results section. | We revised the manuscript to address this concern by relocating the “Study Selection” subsection to the Methods section, where it now follows the description of search and screening procedures. The “Characteristics of Included Sources of Evidence” subsection is presented in the Results section as a descriptive summary of the included studies, consistent with reporting conventions.  Lines 147–155 and 183–204 |
| Facilitators of Implementation - Each point raised in this subsection needs a little more context and explanation. | We revised the Facilitators section to provide additional context and explanation for each theme, expanding beyond list-style reporting to clarify how these factors operated across implementation contexts and influenced engagement, feasibility, and sustainability.  Lines 275–305 |
| Barriers to Implementation - Each point raised in this subsection needs a little more context and explanation. | We revised the Barriers subsection to provide additional context and explanation for each theme, expanding beyond list-style reporting to clarify how these factors operated across implementation contexts and influenced engagement, feasibility, and sustainability.  Lines 306–339 |
| Discussion: The discussion needs a little more description to flesh out your argument. As it stands, it is too shallow. We need to see your argument on what your review says. | We revised the Discussion to more explicitly articulate the central argument of the review. In addition to summarizing findings, the revised text now clarifies how heterogeneity across study designs, contexts, and delivery modalities should be interpreted, and advances the argument that YMHFA functions as a flexible, context-dependent implementation framework rather than a uniform intervention. These revisions were intended to strengthen the interpretive contribution of the review and clarify its implications for implementation and future research.  Lines 338–408 |
| Conclusion: I think conclusion should come before "Gaps in literature" | Fixed |
| Reviewer #2: Thank you for this research. I found the scoping review of mental health first aid for adolecents timely and informative. The scoping study is well laid out and informative beyond the study itself related to implementation and evaluation of mental health programs. | Thank you for reviewing our manuscript. |
| Reviewer #3: The authors conduct a scoping review of YMHFA programmes for adolescents for research published post 2019. Overall, I think the paper is a good contribution to our understanding on how YMHFA programmes can and are implemented alongside commonly observed outcomes. However, I have a few concerns about the manuscript. | Thank you for reviewing our manuscript. |
| YMHFA is the focus of this paper, yet the introduction on what encompasses YMHFA is abrupt and insufficient. While a full narrative review is not needed here, I think much more detail is nevertheless still needed in the introduction for readers who may be unfamiliar with YMHFA and ALGEE. I would recommend including some specific examples of implementation and outcomes here, as well as an overview of the history of these programmes. | We have revised the Introduction to provide a clearer and more detailed overview of YMHFA, including its historical development, international dissemination, and adoption in the United States. We also expanded and clarified the description of the ALGEE action plan and added concrete examples of common implementation settings and outcome domains to better orient readers who may be unfamiliar with the program.  Lines 39–54 |
| The authors have included a comparison with a former systematic review in the introduction, but it's not sufficient. The authors need to justify if the goal is simply to synthesize post-2019 evidence, in which case an updated systematic review would be better, but instead the authors conduct scoping review, because this field now has new directions or niche articles that cannot be fully evaluated through systematic review. Accordingly, the authors need to provide more justification of why these dimensions needs a scoping methodology from mapping complexity, heterogeneity, emerging domains, or insufficient evaluative coherence etc. Additionally, the authors should identify new developments since 2019, and explicitly state how the scope of this review diverges from the 2019 systematic review. | We appreciate this feedback and agree that additional justification was needed. We have revised the Introduction to more explicitly explain why a scoping review was selected rather than an updated systematic review. Specifically, we clarify that since the 2019 systematic review, the YMHFA literature has expanded to include heterogeneous study designs, implementation-focused evaluations, culturally adapted programs, and virtual or hybrid delivery models, particularly following the COVID-19 pandemic. Given this breadth and variability, a scoping review was the most appropriate approach to map emerging domains and characterize implementation contexts rather than synthesize effect sizes or assess study quality.  Lines 61-80 |
| In the same line, why would the authors conduct a scoping review, instead of a more comprehensive systematic review, if the aim is to “provide a comprehensive understanding of YMHFA’s role in youth mental health support”? This seems to me a question better answered by a systematic review than a scoping review. | We have clarified our use of the term “comprehensive” in the Introduction to indicate breadth of coverage across implementation contexts, delivery approaches, and outcome domains, rather than a comprehensive synthesis of intervention effectiveness. We now more clearly align the stated aims of the review with the scoping methodology by emphasizing mapping and characterization of the literature rather than causal inference.  Lines 67–80 |
| The authors provide a definition of mental health literacy, implementations. I think some examples and citations to back up these definitions would be helpful. Moreover, as these are the basis for coding the articles, I think much more justification is needed to show the importance of these elements to research on the effectiveness of YMHFA programmes. For example, why focus on effectiveness – which is on intended outcomes, instead of a broader area of outcomes – which may include intended and unintended effects? | We agree that further clarification was needed regarding the definitions guiding our review. We have revised the Introduction to more explicitly ground our definitions of mental health literacy, implementation, and effectiveness in existing YMHFA literature. We also clarify that “effectiveness” is used pragmatically to reflect the intended outcomes most commonly assessed in YMHFA evaluations (e.g., knowledge, confidence, attitudes, stigma, and helping behaviors), rather than to denote causal or clinical effectiveness. This framing aligns the analytic focus of the review with how outcomes are reported in the existing literature.  Lines 83-98 |
| I think it is not sufficient that the lead author reviewed findings iteratively without any formal inter-rated reliability procedures. Can there be an actual formal procedure, or at least a formal evaluation on the agreement with an external rater, and can there be more information on how the screening team was calibrated, from the pilot screening phase, training, to standardisation procedures, and in doing so, how were disagreements managed? | We agree that greater transparency regarding screening, calibration, and consistency procedures was needed. We have revised the Methods section to more clearly describe the multi-reviewer screening process, including the pilot screening phase, reviewer calibration, and procedures for resolving disagreements.  Consistent with guidance for scoping reviews, formal inter-rater reliability statistics were not calculated, as the aim of the review was to map the breadth of the literature rather than to assess study quality or produce pooled estimates. We have clarified this rationale in the manuscript and expanded our description of the steps taken to promote consistency and transparency throughout the review process.  Lines 149-164 and 171-176 |
| The authors have included a lot of literature which are very heterogeneous, so you need to analyse how heterogeneity affects interpretation, either in the discussion or results sections. For example, how would RCT vs program evaluation affects strength of evidence? How would school vs community-based implementation produce different barriers/facilitators? Are there any different findings from virtual vs in-person? | We agree that heterogeneity across study designs, settings, and delivery modalities has implications for how findings should be interpreted. We have revised the Discussion to more explicitly examine how variation in study design (e.g., randomized controlled trials versus program evaluations), implementation context (e.g., school-based versus community-based settings), and delivery modality (e.g., in-person versus virtual formats) shapes the strength, scope, and interpretation of the available evidence.  Rather than attempting to formally compare or weight heterogeneous studies, consistent with scoping review methodology, we focus on how these differences influence what can be inferred from the literature and where caution or further research is warranted.  Lines 381-408 |
| For outcomes, I noticed that much of the improvement in outcomes were focused on adults involved in adolescent mental health (schools, community, parents etc.). While this is great, were there any direct impacts on improvements in adolescent participants’ mental health? | We agree that the majority of outcomes reported in the YMHFA literature focus on adults who support adolescents, reflecting the program’s design as a gatekeeper intervention. We have revised the Discussion to more explicitly distinguish between adult-level outcomes (e.g., mental health literacy, confidence, helping behaviors) and direct adolescent-level outcomes.  We also clarify that only a small number of studies assessed outcomes among adolescents themselves, such as help-seeking intentions or perceptions of support, and that evidence regarding direct impacts on adolescent mental health remains limited. We now highlight this as a critical gap in the literature and an important direction for future research.  Lines 429-440 |
| The abstract states that the evidence gathered in this review is from US-based sources, yet in the methods it states that the geographic context was not restricted, but language was also limited to English-based articles. Does that mean that only US-based articles were identified (unlikely since there are studies from Australia and the UK)? Or were there some inconsistencies in between the abstract and methods sections? | We have clarified this point in the Abstract to indicate that, while the search strategy did not restrict geographic context, the included literature was predominantly U.S.-based. This revision resolves the inconsistency between the Abstract and Methods sections. |
| I think including information on sample size in the summary table would be helpful, and basic descriptives like age and gender. This may be especially important since outcomes may differ between adolescents, that are the explicit focus of the review, and adults who support or interact with target adolescents groups and are still included in the review. | Where available, we have added sample size and gender distribution information to the summary table. |
| I think a differentiation needs to be made between peer-reviewed journal articles and grey literature (policy documents) which may not have been peer-reviewed in the summary table. | We have revised the summary table to more clearly distinguish between peer-reviewed journal articles and grey literature sources. This distinction is now explicitly indicated to improve transparency regarding the nature of the evidence base. |
